# Hydrocarbon generation kinetics and history of source rocks in different salinity environments, Dongpu Depression, Bohai Bay Basin, China

**DOI:** 10.1371/journal.pone.0350382

**Published:** 2026-06-02

**Authors:** Chengfu Zhang, Jingyan Liu, Tianwu Xu, Jianhong Yang, Lishuang Lv

**Affiliations:** 1 Research Institute of Exploration and Development, Zhongyuan Oilfield, SINOPEC, Puyang, Henan, China; 2 School of Energy Resources, China University of Geosciences(Beijing), Beijing, China; Dawood University of Engineering and Technology, PAKISTAN

## Abstract

The Dongpu Depression is a typical petroliferous depression characterized by remarkable variations in sedimentary salinity. To investigate hydrocarbon generation characteristics of source rocks in different salinity environments, 20 source rock samples were collected and analyzed for organic geochemical. Combining trace elements, Three representative samples were further selected for gold-tube pyrolysis experiments to obtain hydrocarbon generation kinetic parameters. Integrating constraints from kinetic parameters and thermal history, basin modeling was conducted to systematically compare the hydrocarbon generation characteristics of source rocks in different salinity environments. Results show that activation energy for gaseous hydrocarbon generation is widely distributed, while that for liquid hydrocarbon generation is relatively concentrated. Hydrocarbon yields follow the order: saline environment (SE)> brackish environment (BE)> freshwater environment (FE). High-quality source rock intervals with hydrocarbon generation intensity exceeding 2 × 10⁶ t/km² are recognized as key targets for subsequent oil and gas exploration in the region. This study provides valuable implications for hydrocarbon exploration and resource assessment in comparable saline lacustrine basins.

## 1. Introduction

The global distribution of oil and gas resources is closely associated to source rocks developed in environments of varying salinity. Brackish to saline environments commonly host high-quality source rocks [1] and account for the vast majority of global oil and gas reserves [2–6]. Freshwater to brackish environments can also develop excellent source rocks capable of forming giant oil and gas fields with billion-ton reserves [7–9]. In addition, evaporites formed in hypersaline conditions typically act as effective cap rocks for large oil and gas accumulations [10].

However, the influence of contrasting salinity conditions on hydrocarbon generation have not been thoroughly discussed in prior research. Since Tissot et al. (1974) proposed the kinetic model for kerogen hydrocarbon generation [11], Snowdon and Powell (1982, 1991) introduced the concept of immature oil and condensate and modified the hydrocarbon generation model for terrestrial organic matter [8,12]. With ongoing in-depth research, significant differences have been identified in hydrocarbon generation models among source rocks formed in different salinity environments [13–17]. Notably, Cao et al. (2025) established a bimodal hydrocarbon generation model for kerogen in alkaline lacustrine settings [18]. These studies highlight the importance of investigating source rock hydrocarbon generation across varied salinity environments.

Gold-tube pyrolysis experiments for hydrocarbon generation effectively elucidate source rock hydrocarbon generation characteristics. Inclusion of water during experiments can enhance hydrocarbon yields, and inorganic minerals exert catalytic effects on hydrocarbon generation [19–28]. To apply experimental data to geologic settings, Ungerer and Pelet (1987) presented a method to extrapolate the kinetics of oil and gas generation from experiments to sedimentary basins [28]. Sweeney and Burnham (1990) proposed an evaluation of a simple model of vitrinite reflectance based on chemical kinetics. On this basis, Pepper and Corvi (1995) established kinetic curves for hydrocarbon generation under different depositional conditions by integrating experimental and field data [29]. Using basin modeling, hydrocarbon generation kinetics were linked with expulsion processes, enabling quantitative evaluation of hydrocarbon generation at the basin scale. Nevertheless, kerogen formed in different environments shows distinct hydrocarbon generation kinetic parameters [30]. Source rocks commonly undergo multiple episodes of thermal evolution [31]. Therefore, it is necessary to investigate source rock hydrocarbon generation in different environments, with consistent tectonic setting, and thermal history constraints.

The Cenozoic Bohai Bay Basin in China experienced complex thermal evolutionary events, with source rocks deposited in different environments within the Shahejie Formation [32]. The Dongpu Depression is a secondary tectonic unit in the southern part of the Bohai Bay Basin. It contains source rocks formed in freshwater environment(FE), brackish environment(BE), and saline environment(SE) [10,33–35]. In contrast to other depressions in the Bohai Bay Basin, the Dongpu Depression is characterized by halite deposits, overpressure, and high thermal maturity favorable for hydrocarbon generation [36,37]. Thus, the Dongpu Depression provides an ideal natural laboratory to study hydrocarbon generation of source rocks across different salinity environments.

The Dongpu Depression has seen more than 50 years of petroleum exploration. Traditionally, the SE has been considered to hold considerable oil and gas exploration potential. By contrast, although the FE and BE cover a much wider area, they are generally thought to have relatively limited exploration potential [10,33,35]. Yet, recent investigations across the Bohai Bay Basin have identified high-quality source rocks in both FE and BE [9]; These discoveries have also led to a series of major breakthroughs in oil and gas exploration [32,38–41]. This indicates that both FE and BE may also have considerable exploration potential. However, comparative studies on the hydrocarbon generation potential of source rocks formed under contrasting paleoenvironmental conditions remain limited. Against this background, this study aims to obtain hydrocarbon generation kinetic parameters via gold-tube pyrolysis experiments. Combined with the constrained thermal history, these parameters are applied to basin modeling to quantitatively compare the hydrocarbon generation histories of FE, BE, and SE source rocks in the Dongpu Depression.

## 2. Geological setting

**Fig 1:**
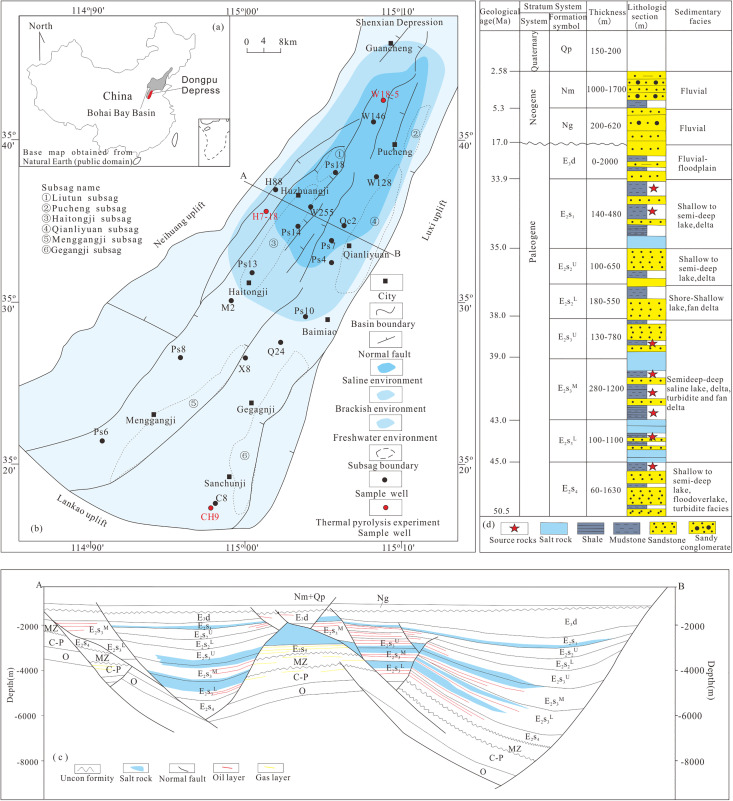
(a) Geographical location of the Dongpu Depression; (b) Tectonic units,Sedimentary environment, Well positions in the Dongpu Depression; (c) Representative cross section (AB) showing the basic geological framework; (d) Generalized Paleogene stratigraphy of the Dongpu Depression.

The Dongpu Depression is a Cenozoic rift basin located on the southern margin of the Bohai Bay Basin, eastern China (Fig 1a). It covers an area of approximately 5300 km² [18,42] (Fig 1b) and reaches a maximum burial depth of about 10,000 m (Fig 1c). Since the Eocene, a thick sedimentary succession has been deposited, including the Shahejie Formation (Members E_2_s_4_, E_2_s_3_, E_2_s_2_, and E_2_s_1_), Dongying Formation, Guantao Formation, Minghuazhen Formation, and Pingyuan Formation [34,38,43](Fig 1d).

The third member of the Shahejie Formation (E_2_s_3_) hosts the major source rocks in the Dongpu Depression [43,44]. Three salt intervals with thicknesses of about 200 m, 260 m, and 200 m are developed within E_2_s_3_ [37,45]. Measured formation pressures range from 10 to 50 MPa [46], indicating widespread overpressure [36]. The second member of the Shahejie Formation (E_2_s_2_) serves as the main reservoir for conventional oil and gas [33]. A thick salt layer in the lower part of the first member of the Shahejie Formation (E_2_s_1_) acts as a regional cap rock [44] (Figs 1c and 1d).

**Fig 2:**
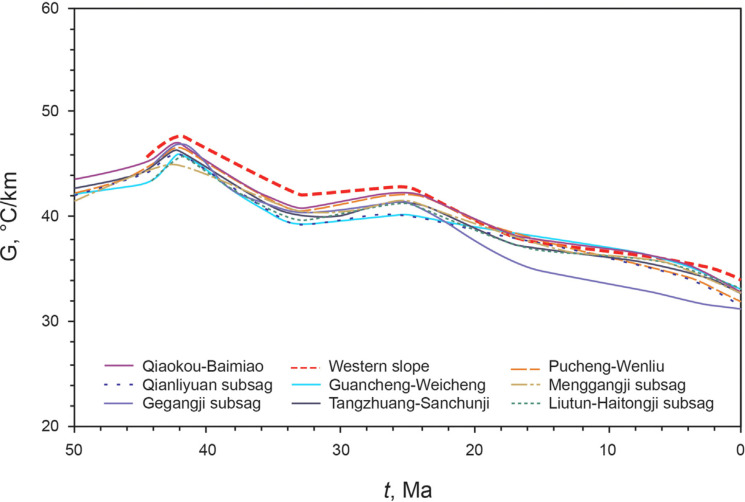
Thermal gradient evolution history of the Dongpu Depression [31].

The Dongpu Depression underwent multiple episodes of Cenozoic tectonic activity. Its thermal evolution can be divided into four stages [31] (Fig 2): (1) Initial rift stage (50–42 Ma): The Lanliao fault zone was highly active, and the geothermal gradient increased. (2) Rapid rift stage (42–33 Ma): Large-scale faulting occurred within the depression, leading to rapid basin expansion and a sharp decrease in the geothermal gradient. (3) Late rift stage (33–27 Ma): Basement faulting gradually weakened, and the geothermal gradient increased. (4) Subsidence stage (27–0 Ma): The Dongying Movement caused erosion of the Dongying Formation [47,48], and the geothermal gradient decreased. Since the Neogene, the Dongpu Depression has entered a stage of slow subsidence, with no increase in the geothermal gradient [31].

## 3. Samples, experiments and methods

Field sampling and core access in the Dongpu Depression were approved by the Zhongyuan Oilfield Company, Sinopec, China. All samples were obtained from existing well cores for academic research. No fieldwork was conducted in protected or restricted areas.

### 3.1. Samples

**Fig 3:**
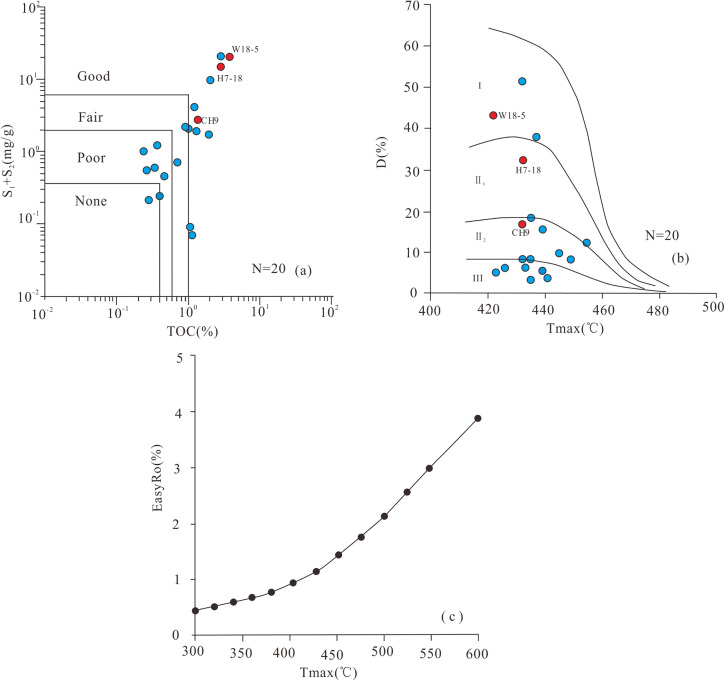
The chart of organic geochemical evaluation of source rocks in different environments. **(a)** The abundance of organic matter of source rocks; **(b)** Organic matter type of source rocks; **(c)** The most method way to calculate Ro in confined gold-tube pyrolysis experiment.

Twenty coring wells were selected covering different salinity environments in the Dongpu Depression (Fig 1b). Guided by centimeter-scale core observations and sedimentary facies analysis, 20 samples were collected from these 20 coring wells. A total of 20 samples were analyzed for organic geochemical characteristics. Based on the experimental results (Table 1, Fig 3), combined with salinity environments and trace element data (Table 2) [34], three representative samples from proven reserve areas were selected for gold-tube pyrolysis experiments. These samples (W18-5, H7-18, and CH9) represent SE, BE, and FE, respectively. All samples are characterized by low thermal maturity and meet the experimental requirements (Table 1).

**Table 1:** Geochemical data of 20 source rock samples in different salinity environments, Dongpu Depression.

Different salinity environment	Sample number	Well	Strata	Depth (m)	Lithology	TOC(%)	Tmax(°C)	S_1_(mg/g)	S_2_(mg/g)	HI(mg/g)	Pc(%)	D(%)	Ro(%)	Remark
SE	1	Ps18	E_2_s_3_^M^	4236.59	Dark gray shale	0.38	435.1	0.52	0.66	143.45	0.09	18.23	1.18	
	2	W18-5	E_2_s_3_^U^	2660.91	dark gray shale	3.81	422.1	6.96	13.17	639.28	1.68	43.09	0.47	*
	3	W146	E_2_s_4_^U^	2838.91	Dark gray shale	2.89	432.0	0.57	19.86	601.78	1.09	51.34	0.52	
	4	Ps14	E_2_s_3_^M^	3921.41	Black mudstone	1.06	439.1	0.69	1.29	151.30	0.16	15.43	1.13	
	5	W255	E_2_s_3_^M^	4588.59	Dark gray shale	1.97	445.0	0.16	1.56	147.35	0.12	9.57	1.25	
	6	Ps7	E_2_s_3_^M^	3510.85	Dark gray shale	1.24	435.0	0.74	3.32	41.47	0.18	8.12	1.14	
	7	W128	E_2_s_3_^M^	3669.30	Dark gray mudstone	0.34	441.0	0.02	0.57	36.12	0.01	3.51	1.02	
	8	Qc2	E_2_s_4_^U^	4084.02	Dark gray mudstone	0.94	364.0	1.18	0.98	104.26	0.18	19.07	1.46	
BE	9	Ps13	E_2_s_3_^U^	4904.10	Black shale	0.71	432.4	0.07	0.63	139.40	0.06	8.24	1.51	
	10	Ps10	E_2_s_3_^L^	4560.00	Dark gray mudstone	0.46	449.0	0.30	0.14	30.43	0.04	7.94	1.24	
	11	Ps4	E_2_s_3_^L^	5107.80	Dark gray shale	1.13	557.8	0.01	0.06	5.15	0.01	0.52	1.53	
	12	H88	E_2_s_3_^L^	1455.14	Dark gray shale	2.02	437.0	0.28	9.34	580.65	0.79	37.84	0.31	
	13	H7-18	E_2_s_3_^L^	2338.63	grey brown shale	2.85	432.3	1.27	13.21	461.69	1.22	32.15	0.55	*
FE	14	Ps6	E_2_s_1_	3377.00	Dark gray mudstone	0.29	433.0	0.08	0.13	44.82	0.02	6.01	1.02	
	15	M2	E_2_s_1_	2808.00	Dark gray mudstone	0.27	426.0	0.01	0.53	41.32	0.02	5.93	0.45	
	16	Ps8	E_2_s_3_^M^	4586.21	Dark gray shale	1.31	454.4	0.71	1.22	113.01	0.16	12.23	1.36	
	17	CH9	E_2_s_3_^U^	2500.13	Dark gray mudstone	1.37	432.3	0.09	2.66	194.89	0.23	16.67	0.53	*
	18	X8	E_2_s_3_^M^	3156.86	dark gray mudstone	0.4	439.2	0.01	0.23	61.14	0.03	5.18	0.86	
	19	Q24	E_2_s_3_^M^	3746.40	Dark gray mudstone	0.24	435.0	0.01	1.00	21.36	0.01	3.17	1.66	
	20	C8	E_2_s_4_^U^	3500.80	Dark gray mudstone	1.07	423.0	0.04	0.05	61.21	0.13	4.98	1.25	

Note: TOC, Total organic carbon; Tmax, peak temperature of pyrolysis; S_1_, free hydrocarbon; S_2_, pyrolyzed hydrocarbon; HI, hydrogen index; Pc, available carbon; D, Degradation rate; Ro, Vitrinite reflectance; * is the sample for gold-tube pyrolysis experiments.

**Table 2:** The indicators representing the paleosalinity for W18-5, H7-18 and CH9, respectively [34].

Samples	B (μg/g)	Ba (μg/g)	Ga (μg/g)	Sr (μg/g)	B/Ga	Sr/Ba
W18-5	152.21	366.60	27.57	1710.00	5.52	4.66
H7-18	103.50	308.40	26.65	1003.00	4.38	3.25
CH9	96.98	382.60	28.39	852.40	3.42	2.23

### 3.2. Organic geochemistry experiments

Pyrolysis experiments were conducted using a Rock-Eval 6 Plus instrument. Samples were heated in a helium atmosphere to yield the S_1_ and S_2_ fractions: S_1_ refers to free hydrocarbons released at 300 °C, and S_2_ corresponds to pyrolytic hydrocarbons liberated between 300 °C and 600 °C. Tmax is defined as the temperature at the maximum S_2_ peak. Total organic carbon (TOC) contents of the samples were determined with a Leco CS230 carbon-sulfur analyzer.

The Vitrinite reflectance (Ro) measurements were conducted using a CRAIC CoalPro instrument. Samples were first crushed to ≤ 1 mm particle size, and air-dried to constant weight at 40 °C. The dried powders were blended with epoxy resin at a volume ratio of 1:1–1:2, then vacuum-cured to form cylindrical pellets with a diameter of approximately 25 mm. The pellets were sequentially ground with 240# to 2000# grit sandpapers for coarse grinding, followed by fine polishing with an alumina polishing agent (≤ 0.05 µm) to attain a mirror-smooth surface. The final polished section was free of scratches, air bubbles, and resin depressions, with a surface roughness (Ra ≤ 0.05 µm). Instrument calibration was performed using certified reflectance standards under 546 nm green light in oil-immersion reflection mode. The polished pellets were mounted on the sample stage and coated with immersion oil before testing. Focusing was achieved using a 50 × objective lens, and reflectance was measured at a minimum of 100 homogeneous vitrinite points to collect robust datasets. Outliers were excluded prior to data processing; the mean values, standard deviations, and frequency histograms were then calculated. Test results were reported following standardized protocols to ensure data accuracy and repeatability.

### 3.3. Confined gold-tube pyrolysis experiments

#### 3.3.1. Gold-tube pyrolysis experiments.

Gold-tube pyrolysis experiments were performed at the Guangzhou Institute of Geochemistry, Chinese Academy of Sciences. A closed gold-tube system was used to simulate hydrocarbon generation from source rocks, with an external fluid pressure of 100 MPa and a maximum heating temperature of 600 °C. This study focused on anhydrous, mineral-free kerogen experiments. Raw samples were ground to a 100-mesh particle size, then treated with hydrochloric acid (HCl) and hydrofluoric acid (HF) to remove carbonate and silicate minerals for kerogen purification. The purified kerogen was neutralized with distilled water, separated via heavy liquid flotation, and dried in an oven at 100 °C for 24 h. All pyrolysis runs were conducted in gold-tube reactors. Kerogen samples were sealed into gold tubes under argon (Ar) protection, and the tubes were closed by arc welding. Each sealed tube was weighed to verify airtightness and ensure no leakage. Sealed gold tubes were placed into autoclaves, and high-pressure pumps were used to inject water into the autoclaves. Elastic deformation of the gold tubes applied targeted pressure to the samples, which was set to 50 MPa and maintained for 30 min, with pressure fluctuations of less than 0.5 MPa. Each sample was heated from room temperature to 250 °C within 10 h, and further heated from 250 °C to 600 °C at two heating rates: 2 °C/h and 20 °C/h. Thirteen temperature points were set for each heating curve, with a temperature interval of ≤ 24 °C. Temperature fluctuation was controlled below 1 °C, and the temperature difference between individual autoclaves was less than 1 °C.

#### 3.3.2. Gas analysis.

Following pyrolysis, gold tubes containing samples were placed into a vacuum system to release generated gases. The vacuum system was coupled online with an Agilent 7890N gas chromatograph (GC), and gas sampling was performed using a vacuum sampling loop. This single injection enabled simultaneous analysis of gases (C_1_–C_5_) hydrocarbon gases, as well as CO, CO₂, H₂, N₂, and O₂.

#### 3.3.3. Liquid hydrocarbons.

After gas analysis, light oil (C_6_–C_14_) diffused into the vacuum glass tube were collected using liquid nitrogen-cooled online vials. Once removed, dichloromethane solvent was immediately injected into the vials. The gold tubes were then retrieved from autoclaves, cut open with the residual samples retained inside, and placed into the same vials. Ultrasonic treatment was conducted for 1 min to fully dissolve generated oils into the solvent, which effectively minimized the loss of light oil. An aliquot (1 mL) of the upper clear supernatant was taken from the 4 mL sample vial and transferred into a 2 mL vial for chromatographic analysis via an auto-sampler. An Agilent 7890N GC equipped with an HP-5 column (30 m × 0.32 mm × 0.25 μm) was used for liquid hydrocarbon analysis. Chromatographic conditions were set as follows: injector temperature of 290 °C; initial oven temperature of 40 °C held for 5 min, then ramped to 290 °C at 4 °C/min and held for 10 min. Deuterated C_24_ was employed as an internal standard for quantitative analysis of light oil (C_6_–C_14_). Quantification of C_6_–C_14_ saturated hydrocarbons and aromatics was performed via GC peak integration. Although full-oil chromatograms were obtained, non-hydrocarbons and asphaltenes in the Heavy oil (C_14+_) fraction are undetectable by GC. Thus, only light oil yields were calculated from chromatograms, and Heavy oil fractions were quantified via extraction, filtration, and gravimetric analysis. Dichloromethane was used as the extraction solvent, and TEFLON organic membranes with a pore size of 0.45 μm were applied for filtration.

#### 3.3.4. Calculation method of hydrocarbon generation kinetic parameters.

Experimental data processing and kinetic simulation calculations were performed using the dedicated Kinetics software developed by Lawrence Livermore National Laboratory (USA). This software applies a parallel first-order reaction model for hydrocarbon generation. the activation energy and frequency factor for each kinetic reaction were obtained via simulation in the Kinetics software.

### 3.4. Methodology for modeling hydrocarbon generation history based on thermal history and kinetic parameters

Key parameters for thermal history analysis include lithology type, geothermal gradient, terrestrial heat flow, petrophysical thermal data, stratigraphic division, stratigraphic age, and erosion amounts during major geological periods. Data on erosion amounts, stratigraphic division, and lithology types for key geological periods were provided by the Zhongyuan Oilfield. Erosion amounts and rock thermal conductivity for each geological period are listed in Table 3, and basal ages of each stratigraphic unit are shown in Fig 1d. Hydrocarbon generation kinetic parameters and thermal history were used as constraints to optimize the temperature variation rate. Combined with source rock geochemical parameters (Table 4), basin modeling software was applied to simulate hydrocarbon generation characteristics of kerogen in different salinity environments.

**Table 3:** Rock thermal conductivity for strata in the Dongpu Depression [31].

Strata	Lithology	Sample No.	Rock thermal conductivity, W/(m.K)	Average rock thermal conductivity, W/(m.K)	Stratum thermal conductivity, W/(m.K)
E_3_d	Siltstone	3	1.42-2.79	2.16	2.20
	Mudstone	1	2.22	2.22	
E_2_s_1_	Limestone	1	2.05	2.05	1.85
	Siltstone	1	2.12	2.12	
	Mudstone	2	1.48-2.08	1.78	
	Salt rock	1	2.67	2.67	
	Dolomite	1	1.95	1.98	
E_2_s_2_	Siltstone	68	1.36-3.06	2.16	2.02
	Mudstone	38	1.25-2.77	1.97	
E_2_s_3_^U^	Dolomite	1	2.26	2.26	1.76
	Siltstone	26	1.34-2.48	1.88	
	Mudstone	33	1.12-2.47	1.72	
	Salt rock	1	2.49	2.49	
E_2_s_3_^M^	Sandstone	3	1.07-3.14	2.26	1.95
	Siltstone	39	1.58-3.13	2.21	
	Mudstone	28	1.16-2.64	1.85	
E_2_s_3_^L^	Siltstone	15	1.47-3.22	2.27	2.01
	Mudstone	13	1.27-2.42	1.84	
	Salt rock	1	2.51	2.51	
E_2_s_4_	Sandstone	3	1.94-2.46	2.26	2.18
	Siltstone	19	1.34-3.12	2.40	
	Mudstone	9	1.60-2.49	2.09	

**Table 4:** Organic matter abundance of source rocks in the third member of the Shahejie Formation, Dongpu Depression [44].

Structural units	TOC(%)	Chloroform asphalt “A”(%)	HC(ppm)	S_1_ + S_2_(mg/g).	Evaluation
Gegangji subsag	0.04-3.85 0.40(508)	0.0013-0.7160 0.0409(157)	0.75-1364.52 56.72(486)	0.001-16.440 0.683(486)	Poor-non source rock
Qianliyuan subsag	0.02-6.51 0.61(2753)	0.0002-1.4153 0.1267(574)	1.33-50820.90 1941.61(1487)	0.002-61.230 2.339(1487)	Medium-good source rock
Menggangji subsag	0.04-1.31 0.40(170)	0.0013-0.1681 0.0278(24)	1.08-1784.50 352.07(92)	0.001-2.150 0.424(92)	Poor-non source rock
Haitongji – Liutun subsag	0.04-8.51 0.80(978)	0.0004-1.9308 0.1776(284)	1.08-56135.00 2272.82(576)	0.001-68.450 2.738(576)	Medium-good source rock

Note: TOC, Total organic carbon; HC, Total hydrocarbon content; S_1_ + S_2_, Hydrocarbon generation potential.

## 4. Results

### 4.1. Organic matter abundance, type and thermal maturity

Vitrinite reflectance (Ro) values of the 20 samples ranged from 0.31% to 1.66%, with an average of 1.02%. Specifically, Ro values for samples from SE different between 0.47% and 1.42% (average = 1.02%); those from BE ranged from 0.31% to 1.53% (average = 1.00%); and values for FE samples spanned 0.45% to 1.66% (average = 1.02%).

Total organic carbon (TOC) contents of the 20 samples fell within the range of 0.24%–3.81% (Table 1), and 13 samples were classified as moderate to good source rocks (Fig 3a). Most source rock samples were characterized by Type Ⅱ_2_ and Type Ⅲ kerogen. The three samples selected for thermal simulation (W18-5, H7-18, and CH9) represented Type I, Type Ⅱ_1_, and Type Ⅱ_2_ kerogen, respectively (Fig 3b).

### 4.2. Hydrocarbon yields

**Fig 4:**
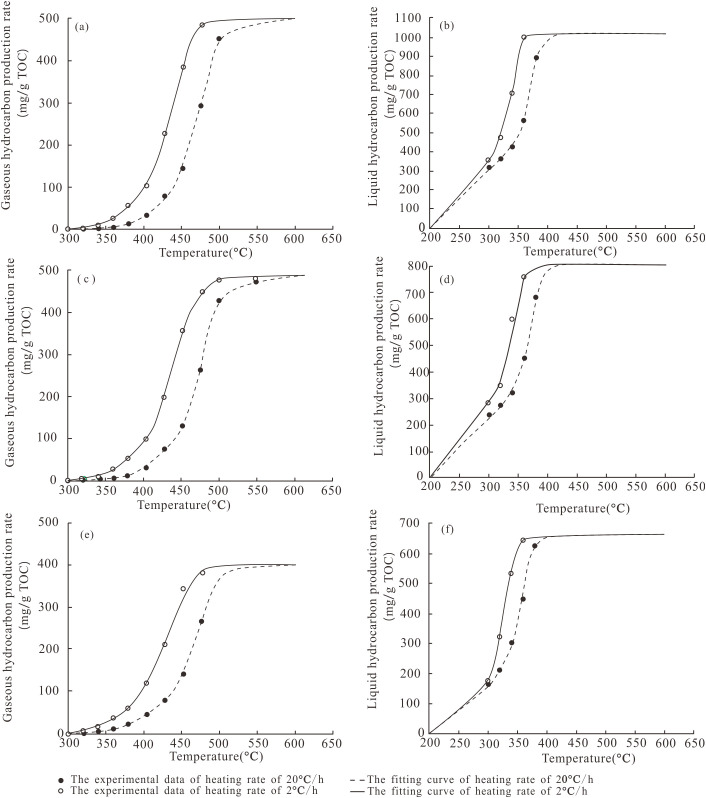
Hydrocarbon generation rates of source rocks from different salinity environments in the Dongpu Depression. **(a)** and **(b)** Well W18-5 in the SE; **(C)** and **(d)** Well H7-18 in the BE; **(e)** and **(f)** Well CH9 in the FE.

Fig 4 and Table 5 summarize the cumulative yields of gaseous hydrocarbons (C_1_-C_5_) and liquid hydrocarbons (C_6+_) for the three samples at the two heating rates (2 °C/h and 20 °C/h). Slow heating (2 °C/h) achieved peak cumulative hydrocarbon yields earlier than rapid heating (20 °C/h).

**Table 5:** Alkane gas and oil yields from closed gold-tube pyrolysis experiments of kerogen from the Dongpu Depression.

Sample ID	Temperature(°C)	Heating rate (°C/h)	Easy%Ro(%)	Yeild(mg/g TOC)					Dryness (%)
				Methane	Ethane	Propane	Hydrocarbon gas	Oil	
W18-5–1	300.0	20	0.44	0.3	0.0	0.0	0.4	319.7	81.4
W18-5–2	320.0	20	0.51	0.6	0.1	0.1	0.9	361.4	74.4
W18-5–3	340.0	20	0.59	1.4	0.3	0.2	2.0	424.7	69.7
W18-5–4	360.0	20	0.68	3.2	1.1	0.8	5.3	562.7	59.1
W18-5–5	380.0	20	0.77	5.6	2.6	2.0	11.3	890.6	49.7
W18-5–6	404.0	20	0.92	12.2	8.1	7.7	34.1	914.5	35.8
W18-5–7	428.0	20	1.15	25.9	19.2	18.4	79.0	726.2	32.8
W18-5–8	452.0	20	1.42	43.8	34.1	35.3	145.0	490.8	30.2
W18-5–9	476.0	20	1.75	78.1	70.2	80.2	294.3	258.5	26.5
W18-5–10	500.1	20	2.13	132.6	121.7	129.1	451.8	172.0	29.4
W18-5–11	524.9	20	2.54	205.5	163.8	129.1	531.2	103.7	38.7
W18-5–12	548.4	20	2.99	278.8	154.4	52.3	492.3	73.9	56.6
W18-5–13	599.7	20	3.87	405.6	61.6	1.1	468.6	18.7	86.6
W18-5–14	299.3	2	0.56	0.9	0.2	0.1	1.2	357.9	74.5
W18-5–15	319.4	2	0.65	1.9	0.5	0.3	2.8	469.3	66.7
W18-5–16	339.5	2	0.75	4.8	2.1	1.5	9.1	707.0	53
W18-5–17	359.8	2	0.86	10.7	6.8	5.5	26.5	1000.5	40.3
W18-5–18	379.5	2	1.04	19.4	14.3	13.0	56.5	787.5	34.3
W18-5–19	403.9	2	1.31	37.2	26.5	24.2	104.7	631.4	35.5
W18-5–20	427.9	2	1.63	67.3	54.1	59.7	227.7	334.6	29.6
W18-5–21	452.1	2	2.02	113.6	96.7	104.9	386.3	166.4	29.4
W18-5–22	477.5	2	2.49	174.5	149.1	121.0	485.0	122.0	36.0
W18-5–23	500.0	2	2.91	254.1	160.7	74.9	500.4	91.4	50.8
W18-5–24	524.2	2	3.38	342.3	132.5	14.1	490.1	29.7	69.9
W18-5–25	548.0	2	3.80	412.5	77.1	1.5	491.4	14.6	84.0
W18-5–26	599.7	2	4.45	479.2	3.8	0.1	483.1	1.0	99.2
H7-18–1	300.0	20	0.44	0.4	0.1	0.1	0.5	238.8	68.0
H7-18–2	320.0	20	0.51	0.7	0.2	0.2	1.2	271.6	61.2
H7-18–3	340.0	20	0.59	1.5	0.7	0.5	2.9	321.5	52.8
H7-18–4	360.0	20	0.68	2.9	1.7	1.3	6.6	450.0	43.4
H7-18–5	380.0	20	0.77	5.2	3.6	2.4	12.3	681.9	42.5
H7-18–6	404.0	20	0.92	11.6	9.3	6.2	29.4	705.4	39.3
H7-18–7	428.0	20	1.15	26.2	22.4	17.0	73.6	570.4	35.6
H7-18–8	452.0	20	1.42	45.3	38.1	31.6	130.1	441.5	34.8
H7-18–9	476.0	20	1.75	88.3	76.2	67.6	262.8	224.1	33.6
H7-18–10	500.1	20	2.13	143.7	121.7	115.2	428.1	100.2	33.6
H7-18–11	524.9	20	2.54	209.1	143.5	97.9	469.9	61.6	44.5
H7-18–12	548.4	20	2.99	287.4	143.1	41.4	475.8	41.3	60.4
H7-18–13	599.7	20	3.87	388.6	33.0	0.6	422.2	10.7	92.0
H7-18–14	299.3	2	0.56	0.9	0.3	0.2	1.6	285.0	60.8
H7-18–15	319.4	2	0.65	1.9	0.9	0.6	3.7	348.9	52.1
H7-18–16	339.5	2	0.75	4.8	3.3	2.1	11.1	598.9	42.8
H7-18–17	359.8	2	0.86	10.4	9.0	6.5	28.7	756.7	36.3
H7-18–18	379.5	2	1.04	19.7	16.8	11.7	53.2	620.0	37.1
H7-18–19	403.9	2	1.31	38.2	29.8	21.7	99.0	494.6	38.5
H7-18–20	427.9	2	1.63	72.4	56.5	47.3	198.2	364.4	36.6
H7-18–21	452.1	2	2.02	121.4	100.6	93.2	357.6	149.2	34.0
H7-18–22	477.5	2	2.49	181.8	129.5	106.1	449.8	75.3	40.4
H7-18–23	500.0	2	2.91	273.6	139.4	58.8	478.8	55.7	57.1
H7-18–24	524.2	2	3.38	358.9	102.5	7.8	469.7	27.8	76.4
H7-18–25	548.0	2	3.80	439.6	41.6	0.8	482.1	11.6	91.2
H7-18–26	599.7	2	4.45	470.0	2.6	0.1	472.7	0.3	99.4
CH9−1	300.0	20	0.44	0.4	0.1	0.1	0.6	165.0	64.1
CH9−2	320.0	20	0.51	0.8	0.3	0.2	1.4	211.0	56.2
CH9−3	340.0	20	0.59	1.7	0.8	0.7	3.8	303.9	44.4
CH9−4	360.0	20	0.68	3.0	2.1	1.8	8.0	447.4	37.3
CH9−5	380.0	20	0.77	5.7	4.9	4.6	19.7	625.9	28.8
CH9−6	404.0	20	0.92	12.2	11.7	10.2	44.4	660.0	27.6
CH9−7	428.0	20	1.15	22.3	20.0	17.4	78.5	551.8	28.5
CH9−8	452.0	20	1.42	40.4	35.3	32.8	138.3	431.5	29.2
CH9−9	476.0	20	1.75	73.2	64.1	68.9	265.8	237.6	27.5
CH9–10	500.1	20	2.13	129.2	96.2	92.6	363.1	120.0	35.6
CH9–11	524.9	20	2.54	197.3	94.9	54.0	353.8	59.3	55.8
CH9–12	548.0	20	2.99	274.7	50.4	7.7	333.2	28.9	82.4
CH9–13	600.0	20	3.87	367.9	1.6	0.1	369.5	18.6	99.6
CH9–14	299.3	2	0.56	1.1	0.4	0.3	2.1	175.0	53.3
CH9–15	319.4	2	0.65	2.3	1.3	1.1	5.3	322.6	42.4
CH9–16	339.5	2	0.75	4.8	4.0	3.6	15.1	534.5	32.0
CH9–17	359.8	2	0.86	10.1	9.9	8.4	36.8	645.6	27.5
CH9–18	379.5	2	1.04	17.1	15.9	12.9	57.9	600.3	29.6
CH9–19	403.9	2	1.31	36.1	31.3	26.8	118.5	501.5	30.5
CH9–20	427.9	2	1.63	64.9	52.4	50.8	211.3	328.6	30.7
CH9–21	452.1	2	2.02	106.9	84.1	87.2	343.9	118.0	31.1
CH9–22	477.5	2	2.49	176.2	103.7	78.9	382.7	60.7	46.0
CH9–23	500.0	2	2.91	268.2	73.6	19.4	362.5	17.2	74.0
CH9–24	524.2	2	3.38	349.6	25.1	0.3	375.0	12.0	93.2
CH9–25	548.0	2	3.80	370.3	3.2	0.1	373.6	6.9	99.1
CH9–26	599.7	2	4.45	376.0	1.1	0.0	377.0	5.1	99.7

Maximum oil and gas yields gradually emerged as thermal maturity increased (Table 5). At a heating rate of 20 °C/h, peak oil yields of W18-5 (SE), H7-18 (BE), and CH9 (FE) were 914.5 mg/g TOC, 705.4 mg/g TOC, and 660.0 mg/g TOC, respectively. Peak gas yields were 531.2 mg/g TOC, 475.8 mg/g TOC, and 369.5 mg/g TOC, respectively. All kerogen types (Type I/SE, Type Ⅱ_1_/BE, Type Ⅱ_2_/FE) underwent oil generation followed by gas generation. Hydrocarbon yields rose to a peak before declining, with both oil and gas yields following the sequence: Type I kerogen (SE)> Type Ⅱ_1_ kerogen (BE)> Type Ⅱ_2_ kerogen (FE). Natural gas dryness ratios all exhibited an initial decrease followed by an increase (Table 5).

### 4.3. Hydrocarbon generation characteristics of kerogen in different salinity environments

**Fig 5:**
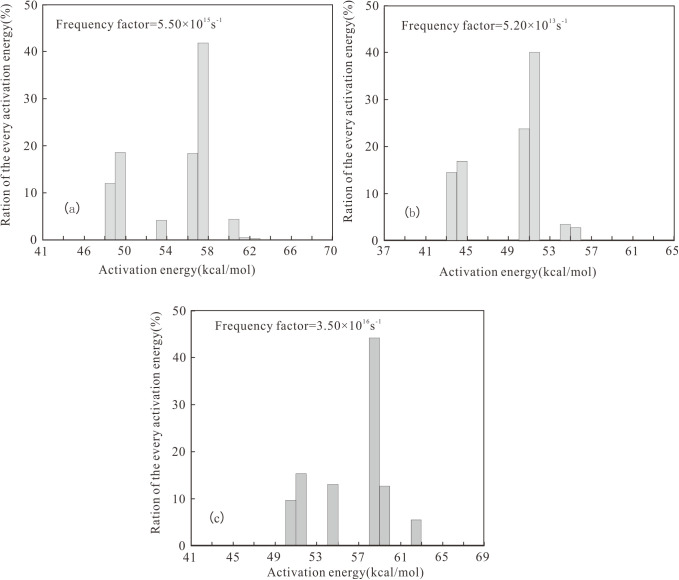
Activation energy distribution of liquid hydrocarbon in different salinity environments of Dongpu Depression. **(a)** Well W18-5 (SE); **(b)** Well H7-18 (BE); **(c)** Well CH9 (FE).

**Fig 6:**
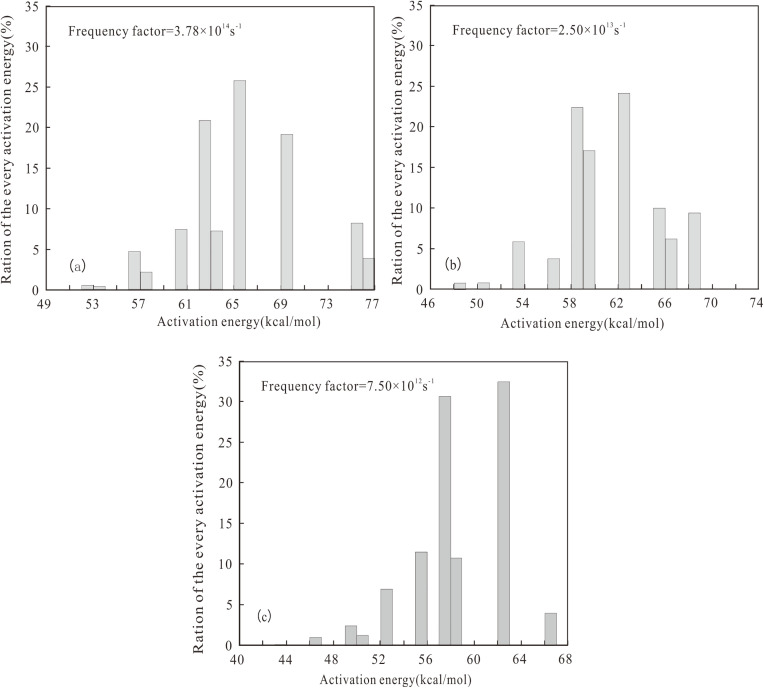
Activation energy distribution of gaseous hydrocarbon in different salinity environments of Dongpu Depression. **(a)** Well W18-5 (SE); **(b)** Well H7-18 (BE); **(c)** Well CH9 (FE).

Kinetic parameters for hydrocarbon generation of kerogen in different salinity environments in the Dongpu Depression are shown in Figs 5–6 and Table 6.

**Table 6:** Kinetic parameters of hydrocarbon generation in different salinity environments of Dongpu Depression.

Salinity environment (Well）	Primary cracking			Secondary cracking			Initial potential of oil(mg/g TOC)	Initial potential of gaseous hydrocarbon(mg/g TOC)	Second potential of gaseous hydrocarbon(mg/g TOC)
	Frequency	the activation energy (kcal/mol)	Frequency factor(s-1)	Frequency	the activation energy (kcal/mol)	Frequency factor(s-1)			
SE(W18-5)	0.123	48	5.5 × 10^15^	0.002	52	3.8 × 10^14^	649.3	231.2	145.9
	0.190	49	5.5 × 10^15^	0.001	53	3.8 × 10^14^			
	0.048	53	5.5 × 10^15^	0.048	56	3.8 × 10^14^			
	0.189	56	5.5 × 10^15^	0.020	57	3.8 × 10^14^			
	0.421	57	5.5 × 10^15^	0.069	60	3.8 × 10^14^			
	0.049	61	5.5 × 10^15^	0.211	62	3.8 × 10^14^			
	0.002	62	5.5 × 10^15^	0.068	63	3.8 × 10^14^			
	0.001	63	5.5 × 10^15^	0.263	65	3.8 × 10^14^			
				0.182	69	3.8 × 10^14^			
				0.075	75	3.8 × 10^14^			
				0.037	76	3.8 × 10^14^			
BE (H7-18)	0.143	43	5.2 × 10^13^	0.008	48	2.5 × 10^13^	500.8	201.9	135.9
	0.167	44	5.2 × 10^13^	0.007	50	2.5 × 10^13^			
	0.234	50	5.2 × 10^13^	0.059	53	2.5 × 10^13^			
	0.398	51	5.2 × 10^13^	0.036	56	2.5 × 10^13^			
	0.033	54	5.2 × 10^13^	0.224	58	2.5 × 10^13^			
	0.026	55	5.2 × 10^13^	0.170	59	2.5 × 10^13^			
				0.241	62	2.5 × 10^13^			
				0.100	65	2.5 × 10^13^			
				0.061	66	2.5 × 10^13^			
				0.093	68	2.5 × 10^13^			
FE (CH9)	0.097	50	3.5 × 10^16^	0.010	46	7.5 × 10^12^	374.4	132.7	77.1
	0.151	51	3.5 × 10^16^	0.025	49	7.5 × 10^12^			
	0.137	55	3.5 × 10^16^	0.013	50	7.5 × 10^12^			
	0.448	58	3.5 × 10^16^	0.067	52	7.5 × 10^12^			
	0.113	59	3.5 × 10^16^	0.121	55	7.5 × 10^12^			
	0.052	62	3.5 × 10^16^	0.313	57	7.5 × 10^12^			
				0.114	58	7.5 × 10^12^			
				0.340	62	7.5 × 10^12^			
				0.043	67	7.5 × 10^12^			

For Type I kerogen (SE), the activation energy for liquid hydrocarbon generation ranged widely from 48 to 62 kcal/mol, with a main peak at 57 kcal/mol and a frequency factor of 5.50 × 10^15^ s^-1^ (Fig 5a). The activation energy for gaseous hydrocarbon generation ranged from 52 to 76 kcal/mol, with a main peak at 65 kcal/mol and a frequency factor of 3.78 × 10^14^ s^-1^ (Fig 6a).

For Type Ⅱ_1_ kerogen (BE), the activation energy for liquid hydrocarbon generation was concentrated between 43 and 55 kcal/mol, with a main peak at 51 kcal/mol and a frequency factor of 5.20 × 10^13^ s^-1^ (Fig 5b). The activation energy for gaseous hydrocarbon generation ranged widely from 48 to 68 kcal/mol, with a main peak at 62 kcal/mol and a frequency factor of 2.50 × 10^13^ s^-1^ (Fig 6b).

For Type Ⅱ_2_ kerogen (FE), the activation energy for liquid hydrocarbon generation was concentrated between 50 and 63 kcal/mol, with a main peak at 58 kcal/mol and a frequency factor of 3.50 × 10^16^ s^-1^ (Fig 5c). The activation energy for gaseous hydrocarbon generation was concentrated between 46 and 67 kcal/mol, with a main peak at 62 kcal/mol and a frequency factor of 7.50 × 10^12^ s^-1^ (Fig 6c).

**Fig 7:**
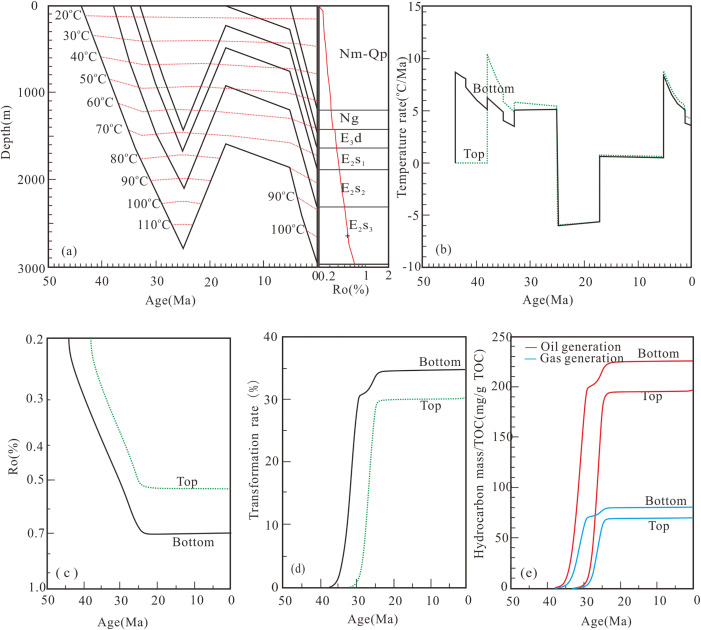
Hydrocarbon generation history of well W18-5 (SE) in the third member of the Shahejie Formation. **(a)** Buried history and thermal history; **(b)** Temperature rate; **(c)** Maturity history; **(d)** Transformation rate; **(e)** Hydrocarbon generation history.

**Fig 8:**
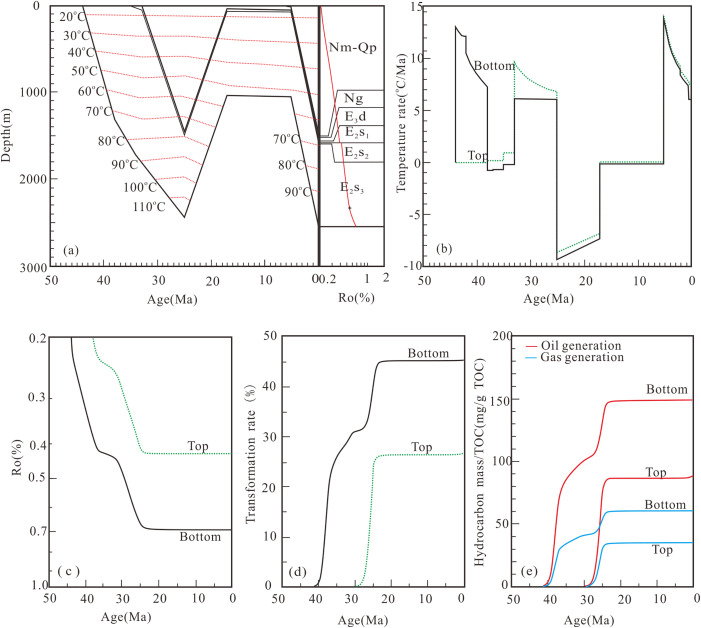
Hydrocarbon generation history of well H7-18 (BE) in the third member of the Shahejie Formation. **(a)** Buried history and thermal history; **(b)** Temperature rate; **(c)** Maturity history; **(d)** Transformation rate; **(e)** Hydrocarbon generation history.

**Fig 9:**
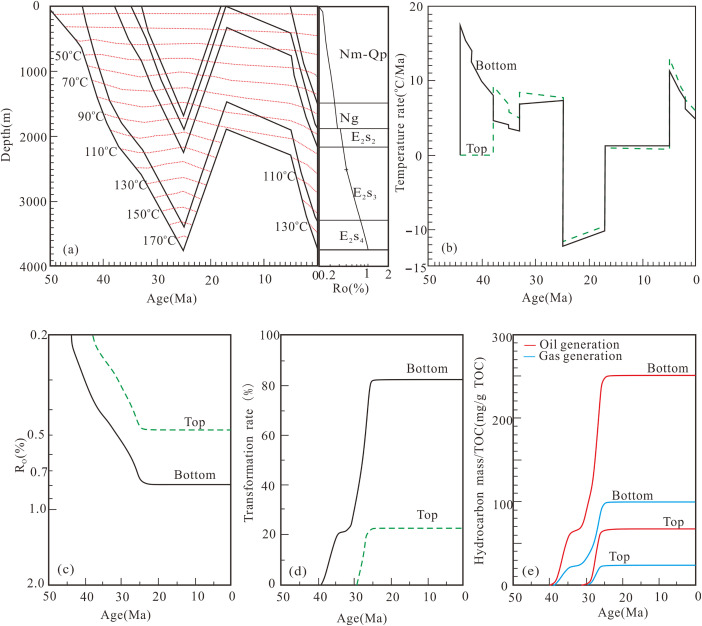
Hydrocarbon generation history of well CH9 (FE) in the third member of the Shahejie Formation. **(a)** Buried history and thermal history; **(b)** Temperature rate; **(c)** Maturity history; **(d)** Transformation rate; **(e)** Hydrocarbon generation history.

Basin-modeled hydrocarbon generation histories for the sampling wells are presented in Figs 7–9.

Hydrocarbon generation from source rocks at the bottom of the third member of the Shahejie Formation (E_2_s_3_) in Well W18-5 (SE) occurred in two stages. The first stage (early deposition of the second member of the Shahejie Formation to early deposition of the Dongying Formation) was marked by rapid hydrocarbon generation and constituted the main generation phase. The second stage (late deposition of the Dongying Formation) saw gradual hydrocarbon generation, peaking at maximum oil and gas yields of 225.9 mg/g TOC and 80.4 mg/g TOC, respectively. Source rocks at the top of E_2_s_3_ in Well W18-5 generated hydrocarbons rapidly from the early to late stages of Dongying Formation deposition, with maximum oil and gas yields of 196.8 mg/g TOC and 69.9 mg/g TOC, respectively (Fig 7).

Hydrocarbon generation from E_2_s_3_ bottom source rocks in Well H7-18 (BE) likewise occurred in two stages. The first stage (from middle E_2_s_3_ deposition to early Dongying Formation deposition) represented the main phase of rapid hydrocarbon generation. The second stage persisted into late Dongying Formation deposition, with peak oil and gas yields of 149.0 mg/g TOC and 60.1 mg/g TOC, respectively. Source rocks at the top of E_2_s_3_ in Well H7-18 generated hydrocarbons rapidly from the early to middle stages of Dongying Formation deposition, with maximum oil and gas yields of 87.5 mg/g TOC and 35.0 mg/g TOC, respectively (Fig 8).

Hydrocarbon generation from E_2_s_3_ bottom source rocks in Well CH9 (FE) was split into two stages. Rapid oil generation took place from middle E_2_s_3_ deposition to early Dongying Formation deposition (the main oil generation period), with peak oil and gas yields of 251.0 mg/g TOC and 98.8 mg/g TOC, respectively. For the E_2_s_3_ top source rocks, the maximum oil and gas yields were 67.3 mg/g TOC and 23.8 mg/g TOC, respectively (Fig 9).

## 5. Discussion

### 5.1. Representativeness of samples

Among the 20 samples, W18-5, W146, H88, H7-18, and CH9 exhibited low Ro values coupled with high TOC contents (Table 1). Continuous sampling was carried out for W18-5, H7-18, and CH9, yielding a total of 41 analyzed samples [34]; Trace elements were also measured for a subset of these samples (Table 2). These results collectively confirm that W18-5, H7-18, and CH9 are representative of the SE, BE, and FE, respectively.

### 5.2. Modeling of hydrocarbon generation history

The E_2_s_3_ interval in the Dongpu Depression underwent multiple episodes of temperature variation (Figs 7b, 8b and 9b), which is consistent with field geological conditions and previous studies [30,31,47]. Following previous studies of the Dongpu Depression [30–31], a constant paleosurface temperature is adopted, which exerts negligible influence on hydrocarbon generation results and main conclusions. Source rocks of the Shahejie Formation record two major episodes of hydrocarbon generation: the Dongying stage (~25 Ma) and the present day (0 Ma), with the Dongying stage characterized by the highest generation intensity. This stage also marks the main period of hydrocarbon expulsion in the Dongpu Depression [10,30,36,47]. Fluid inclusions formed during the Dongying stage are hosted in minerals and primary pores, whereas those formed from the Minghuazhen stage to the present occur mainly in fractures [39,48]. Carbon isotope data for natural gas suggest that gas accumulation in the Dongpu Depression took place at 28.6–25.8 Ma [35].

### 5.3. Hydrocarbon generation potential

**Fig 10:**
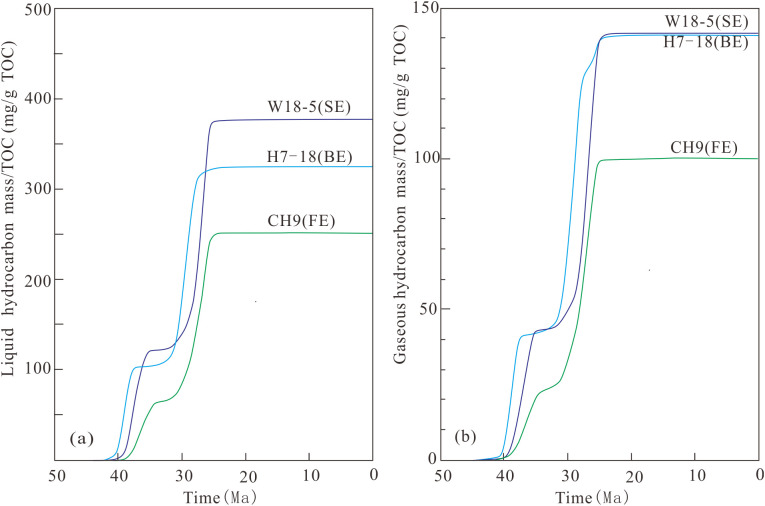
Comparison of hydrocarbon generation potentials of varied salinity environments. **(a)** Liquid hydrocarbon potential; **(b)** Gaseous hydrocarbon potential.

Direct comparison of hydrocarbon generation results from Figs 7–9 is invalid owing to differing burial and thermal histories across distinct structural locations [30,43]. To evaluate the hydrocarbon generation potential of kerogen from different salinity environments, identical parameters were assumed for the three samples, with the exception of depositional environment and kerogen type. Based on paleothermal, basic geological, and geochemical data from Well H7-18 (BE), kinetic parameters and hydrogen indices were adjusted to match those of the other two wells to enable cross-comparison (Fig 10). Results show that liquid hydrocarbon generation potential follows the order SE > BE > FE, whereas gaseous hydrocarbon potential follows SE ≈ BE > FE. This trend is likely attributed to greater sapropelic components in SE relative to BE and FE, as well as higher inertinite content in FE compared with SE and BE [34]. In addition, algal blooms occurred in both SE and BE, promoting the formation of organic-rich shales characterized by high TOC and elevated paleoproductivity [8,10,12,18,34,42].

### 5.4. Hydrocarbon generation evolution

**Fig 11:**
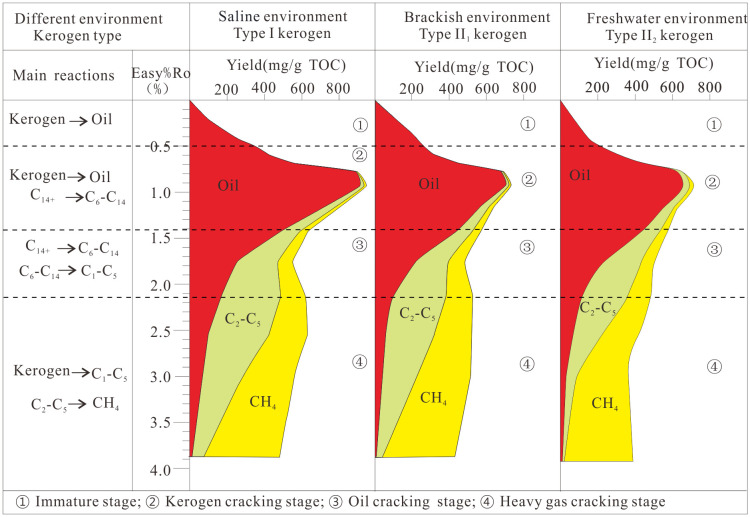
Hydrocarbon generation and evolution model of source rocks in varied salinity environments.

A hydrocarbon generation evolution model for source rocks in different salinity environments was established based on gold-tube pyrolysis experiments (Fig 11).

① When easy%Ro < 0.5% (biogenic gas generation stage): Yields for this stage are inferred from Fig 11 owing to a lack of experimental products, so this stage is not discussed further.

② When 0.5% ≤ easy%Ro < 0.92%: Kerogen generates abundant oil (dominated by C_14+_). Type I (SE) and Type Ⅱ_1_ (BE) kerogens reach peak oil generation at ~0.9% easy%Ro, while Type Ⅱ_2_ (FE) kerogen peaks at ~0.8% easy%Ro. Observed kinetic differences likely reflect combined effects of salinity-related depositional conditions and kerogen properties. Low geothermal gradients, and overpressure may shift the peak oil-generation Ro values, delaying it to as high as 1.5% [13,46,49].

When 0.92% ≤ easy%Ro < 1.42%: Heavy oil (C_14+_) begins to crack into light oil (C_6_-C_14_) and gas. The thermal stability of crude oil is governed by its physical properties and chemical composition [50]. Cracking of light oil (C_6_-C_14_) into gases (C_1_-C_5_) is insignificant prior to the C_6_-C_14_ yield peak [51–53].

③ When 1.42% ≤ easy%Ro < 2.13%: Increasing temperatures drive widespread generation of light oil (C_6_-C_14_) and gases (C_1_-C_5_). Infrared spectroscopy reveals residual short-branched aliphatic structures in kerogen during high to overmature stages [25].

④ When easy%Ro ≥ 2.13%: Large volumes of methane (CH_4_) form, accompanied by minor heavy hydrocarbon gases (C_2_-C_5_) and residual oil. Small quantities of residual oil have been documented in prior studies [18,34,43,54]. Cracking simulations show that crude oil undergoes extensive cracking at 190–210 °C, whereas ethane cracking requires temperatures exceeding >230 °C [55]. Accordingly, residual oil most likely represents unpyrolyzed oil retained from earlier stages. These results show that oil can remain stable through high to overmature stages. This helps form deep and ultra-deep oil reservoirs with efficient expulsion and effective preservation.

### 5.5. Hydrocarbon generation intensity

**Fig 12:**
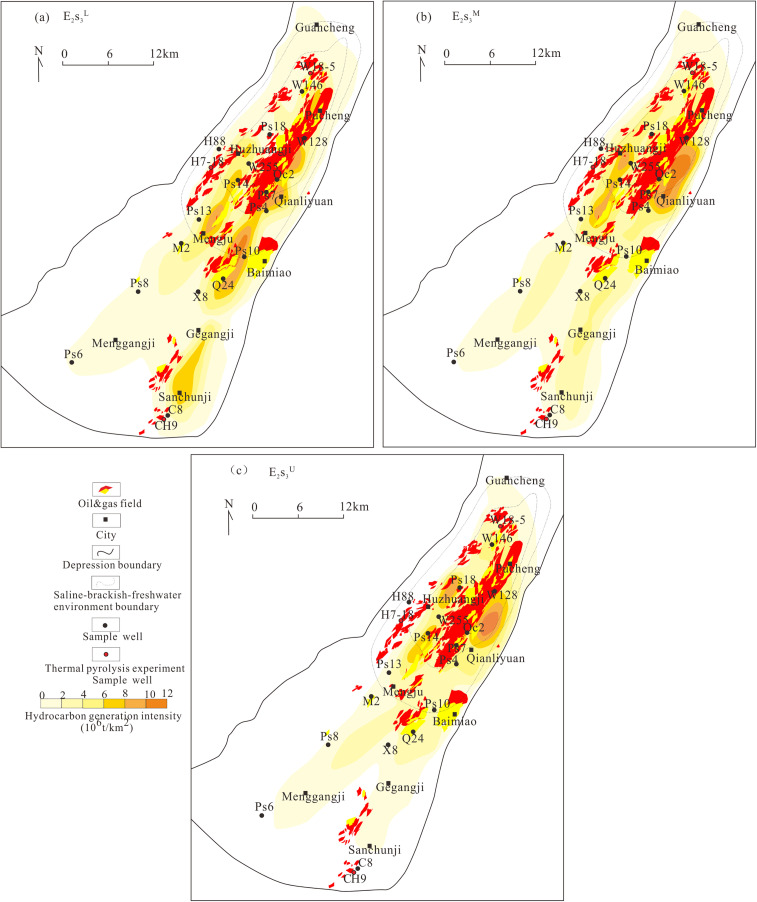
Hydrocarbon generation intensity map of the third member of the Shahejie Formation in the Dongpu Depression. **(a)** Lower third submember of the Shahejie Formation(E_2_s_3_^L^); **(b)** Middle third submember of the Shahejie Formation(E_2_s_3_^M^), **(c)** Upper third submember of the Shahejie Formation(E_2_s_3_^U^).

Hydrocarbon generation intensity of E_2_s_3_ source rocks during the key Dongying stage (25 Ma) (Fig 12) demonstrates that generation intensity is primarily controlled by salinity environment and subsag belt distribution. Sub-sag belts display the highest hydrocarbon generation intensity, followed by slope belts and uplift belts, rendering sub-sags the primary centers of hydrocarbon generation and expulsion [48]. Generation intensity follows the order: SE > BE > FE. The widespread southern FE exhibits comparable exploration potential to the northern SE, with significant exploration breakthroughs recently achieved in FE sub-sags [38]. Areas with generation intensity >2 × 10^6^ t/km^2^ constitute priority targets for future oil and gas exploration.

## 6. Conclusions

(1) The main peak of the activation energy for liquid hydrocarbon generation follows: Type Ⅱ_1_ kerogen (BE) <Type I kerogen (SE) <Type Ⅱ_2_ kerogen (FE). The main peak of the activation energy for gaseous hydrocarbon generation follows: Type Ⅱ_2_ kerogen (FE) = Type Ⅱ_1_ kerogen (BE) <Type I kerogen (SE). Total hydrocarbon yields are highest in SE samples, followed by those from BE and FE.
(2) Hydrocarbon generation of source rocks in different salinity environments can be divided into three distinct stages: kerogen cracking to generate oil during the mature stage; heavy oil (C_14+_) cracking into light oil (C_6_-C_14_) and gas (C_1_-C_5_) at the high-mature stage; and extensive methane generation at the overmature stage.
(3) Hydrocarbon generation intensity in the Dongpu Depression decreases in the sequence of SE > BE > FE. The widespread southern FE exhibits comparable exploration potential to the northern SE. Areas with generation intensity exceeding 2 × 10^6^ t/km^2^ represent the top priority targets for subsequent hydrocarbon exploration.
